# Comparative Metabolomics in Single Ventricle Patients after Fontan Palliation: A Strong Case for a Targeted Metabolic Therapy

**DOI:** 10.3390/metabo13080932

**Published:** 2023-08-09

**Authors:** David Renaud, Sabine Scholl-Bürgi, Daniela Karall, Miriam Michel

**Affiliations:** 1 Fundamental and Biomedical Sciences, Paris-Cité University, 75006 Paris, France; 2 Health Sciences Faculty, Universidad Europea Miguel de Cervantes, 47012 Valladolid, Spain; 3 Fundacja Recover, 05-124 Skrzeszew, Poland; 4 Department of Child and Adolescent Health, Division of Pediatrics I—Inherited Metabolic Disorders, Medical University of Innsbruck, 6020 Innsbruck, Austria; 5 Department of Child and Adolescent Health, Division of Pediatrics III—Cardiology, Pulmonology, Allergology and Cystic Fibrosis, Medical University of Innsbruck, 6020 Innsbruck, Austria

**Keywords:** biventricular, heart failure, nutrition, Fontan, ketones, ketogenic therapy, metabolism, single ventricle

## Abstract

Most studies on single ventricle (SV) circulation take a physiological or anatomical approach. Although there is a tight coupling between cardiac contractility and metabolism, the metabolic perspective on this patient population is very recent. Early findings point to major metabolic disturbances, with both impaired glucose and fatty acid oxidation in the cardiomyocytes. Additionally, Fontan patients have systemic metabolic derangements such as abnormal glucose metabolism and hypocholesterolemia. Our literature review compares the metabolism of patients with a SV circulation after Fontan palliation with that of patients with a healthy biventricular (BV) heart, or different subtypes of a failing BV heart, by Pubmed review of the literature on cardiac metabolism, Fontan failure, heart failure (HF), ketosis, metabolism published in English from 1939 to 2023. Early evidence demonstrates that SV circulation is not only a hemodynamic burden requiring staged palliation, but also a metabolic issue with alterations similar to what is known for HF in a BV circulation. Alterations of fatty acid and glucose oxidation were found, resulting in metabolic instability and impaired energy production. As reported for patients with BV HF, stimulating ketone oxidation may be an effective treatment strategy for HF in these patients. Few but promising clinical trials have been conducted thus far to evaluate therapeutic ketosis with HF using a variety of instruments, including ketogenic diet, ketone esters, and sodium-glucose co-transporter-2 (SGLT2) inhibitors. An initial trial on a small cohort demonstrated favorable outcomes for Fontan patients treated with SGLT2 inhibitors. Therapeutic ketosis is worth considering in the treatment of Fontan patients, as ketones positively affect not only the myocardial energy metabolism, but also the global Fontan physiopathology. Induced ketosis seems promising as a concerted therapeutic strategy.

## 1. Introduction

Children with complex congenital heart disease (CHD) and single ventricle (SV) physiology typically undergo several-step surgical palliation with the aim of a total cavopulmonary connection. In this so-called Fontan circulation, the subpulmonary pump is missing. Instead, the vena cavae are anastomosed directly to the pulmonary arteries, causing elevated systemic venous pressure and chronically decreased cardiac output [1,2,3,4].

Even if the outcome of SV patients is steadily improving, particularly their largest subgroup—i.e., SV patients with aortic atresia and a hypoplastic left heart (HLHS, 40% of cases [5]) and a morphologically right ventricle (RV) serving as the subsystemic ventricle—performs worse than patients with a morphologically left ventricle (LV) [5,6,7,8,9,10,11,12,13,14], particularly with respect to atrioventricular valve failure, impaired ventricular function and/or failure of the Fontan circulation with upstream issues such as liver cirrhosis or protein-losing enteropathy [15,16,17]. Ventricular dysfunction is currently considered to be inevitable for SV patients [18]. In addition to these complications, Fontan patients reveal alterations in their pulmonary, hematologic, immunologic, endocrinologic and metabolic systems [1,19].

Pharmacological interventions for both patients with a failing biventricular (BV) and patients with a failing SV heart currently target either hemodynamics or the neuro-hormonal axis, with conflicting evidence regarding the clinical benefit [20,21,22,23,24]. A more recent axis actively targeted in BV HF therapy is the metabolic axis [25,26,27,28,29,30,31,32,33,34,35,36]. Maintaining ketone metabolism is reported to have a protective effect in hypertrophic and failing BV hearts [37,38,39,40,41]. Keeping in mind the metabolic alterations in the Fontan patient [42,43], metabolism may be a promising therapeutic target also in Fontan patients with a failing SV or Fontan circulatory failure.

Reviewing the literature, we compare the energy metabolism in patients with healthy, or with a failing BV heart with that reported in SV patients after Fontan palliation. Focusing on lipid and ketone metabolism, and focusing on the role of therapeutic ketosis in BV HF, a potential role of metabolism-targeted therapy in Fontan patients will be discussed.

## 2. Cardiac Energy Metabolism

The heart is a biomechanical pump with complex hemodynamics. Ninety percent of the cellular adenosine triphosphate (ATP) is used to sustain the contraction-relaxation of the cardiac muscle [44]. Mitochondria make up one-third of the cardiac myocyte volume [45]. In the whole human body, cardiomyocytes exhibit the highest content in mitochondria, consistent with the fact that the heart is the organ with the highest energy consumption [46]. Thus, it is not surprising that an altered energy state could contribute to HF. The energy starvation model has been proposed as the basis of progressive HF [47,48,49,50], taking into account alterations of the three different stages of ATP production: perturbed substrate consumption; altered oxidative phosphorylation; and reduced energy transfer to ATP-consuming reactions. ATP production is controlled and regulated by a very complex set of transcriptions of metabolically relevant proteins (receptors, regulators, transporters, enzymes) that can be quantified through analysis of metabolites.

### 2.1. Energy Metabolism in the Healthy Heart

*The “omnivore heart” and its diverse substrate consumption.* Cardiomyocytes have the capacity to oxidize fatty acids, carbohydrates, amino acids, ketones, and lactate [51]. The substrate preferences are changing with development. The fetus lives in a hypoxic environment, what was called ‘Everest in utero’ [52], and depends on his mother‘s metabolism. Glucose is the dominant substrate used for ATP production, with a very low level of fatty acids and high level of lactate [53,54]. Change of available substrates, increasing oxygen level and improved cardiac workload drive metabolic maturation. Originally, it was thought that within the first week postnatally, glucose use in the heart drops significantly with fatty acid oxidation rising [55], reaching an adult metabolic pattern. Recent studies hypothesize that transition to fatty acid oxidation may start earlier, from the late gestational period [56]. Due to the surge of oxidative capacity—one third of cardiac myocytes’ volume being mitochondria—60–90% of the energy used for mechanical performance originates from mitochondrial fatty acid oxidation, with the remainder originating from glucose, lactate, and ketone bodies [57,58] (Table 1).

Biosynthesis of ketone bodies is connected to various metabolic pathways such as beta-oxidation, Krebs cycle, sterol biosynthesis, glucose metabolism, and mitochondrial electron transport chain [36,99,100]. Among all organs, the heart tissue exhibits the highest levels of ketolytic enzyme activity, reflecting its ability to use ketone bodies [101]. Nonetheless, under physiological conditions, ketone bodies are not contributing significantly to cardiac metabolism [102], glucose and fatty acids being the predominant substrates [61,62].

Substrate utilization is meticulously regulated, which is mandatory due to the relatively small amount of stored ATP relative to the rates of myocytal ATP consumption [49]. One of the control mechanisms is the glucose-fatty-acid-cycle. Numerous parallel mechanisms regulating the substrate utilization in cardiomyocytes were discovered, such as glucose transporter (Glut) 1, Glut 4, or peroxisome proliferator activated receptors (PPAR) [59,103].

*From fuel to ATP: oxidative phosphorylation.* Oxidative phosphorylation begins with the oxidation of substrates, such as fatty acids and glucose, which are converted into acetyl-coenzyme A (CoA) through pyruvate or beta-oxidation. Acetyl-CoA is fed into the Krebs cycle. Under normal conditions, mitochondrial oxidative phosphorylation matches 90–95% of the myocardial ATP demand with glycolysis filling the gap [61].

*From ATP to contractile work: creatine phosphate reserve of ATP.* The creatine kinase (CK) energy shuttle plays a crucial role in maintaining energy balance in cells with high and fluctuating energy demands, such as cardiomyocytes: It comprises the conversion of creatine to phosphocreatine (and back), which serves as a rapid and reversible energy storage system. This pathway enables the efficient transfer of energy from the mitochondria, where ATP is produced by oxidative phosphorylation, to the cytosol, where it is consumed during contraction.

*Regulation of metabolism through gene expression.* Control and regulation of cardiac metabolism are complex and include overexpression or deletion of metabolically relevant proteins, such as receptors, regulators, transporters, or enzymes.

*Peroxisome proliferator activated receptors.* PPAR are a family of nuclear receptor proteins that play a crucial role in regulating the expression of genes involved in lipid and glucose metabolism, inflammation, and cellular differentiation. PPAR is an abundantly expressed key regulator of cardiac substrate switching [104,105,106,107], including fatty acid oxidation, ketogenesis, and triglyceride synthesis, by upregulation of genes involved in fatty acid metabolism. Activation of PPAR upregulates genes of fatty acid oxidation (fatty acid transport protein/cluster of differentiation 36, malonyl-CoA decarboxylase, carnitine acyltransferase (CPT)-1, medium and long chain acyl-CoA dehydrogenases) [102]. PPAR was downregulated by hypoxia in rats’ hearts [108].

*Adenosine monophosphate-activated protein kinase pathway, GLUT and CPT.* The adenosine monophosphate-activated protein kinase (AMPK) pathway is a critical cellular signaling pathway that plays a crucial role in regulating energy homeostasis in cells. The activation of the AMPK pathway leads to the phosphorylation of numerous downstream targets involved in metabolism, transcription, and protein synthesis. Through this pathway, CPT1, medium-chain acyl-CoA dehydrogenase, cluster of differentiation 36, and fatty acid transport protein 1 are decreased in HF [61]. Inhibition of CPT1 directly inhibits fatty acid oxidation by malonyl-CoA—a phenomenon called reverse Randle effect [105].

*Histone acetylation/deacetylation.* Acetylation is a protein post-translational modification controlling expression and transcription of genes, regulating embryonic development, postnatal fatty acid oxidation maturation, and heart hypertrophy. It enables the cell to quickly and effectively react to cellular stress [109]. Histone acetylation regulates the electrostatic connections between DNA and histones as well as between adjacent nucleosomes within a nucleosomal fiber, which controls transcription [110]. Histone acetylationtransferases (HAT), also known as lysine acetyltransferases, which relax chromatin structure, and histone deacetylases (HDAC) which reverse the HAT process, reduce transcriptional activity and are the main regulators of HAT [111]. HDAC can be divided into two types based on their architectures and patterns of expression [112]. All tissues express class I HDAC. Class II histone deacetylases interact with the MEF2 transcription factor to control fetal cardiac and stress-responsive genes [113,114]. Activity of GLUT1, GLUT4, PDK2, muscle-glycogen synthase, mCPT-1, MCAD, and ACC is higher in the non-failing adult human heart than in the fetal heart. In the failing human heart, those metabolic genes‘ activities decrease to the same levels as in the fetal heart [60].

### 2.2. Energy Metabolism in Biventricular Patients with Congestive Heart Failure

*Disturbed substrate consumption.* Once the heart has reached its metabolic adulthood, the main substrates used for ATP production are fatty acids (60–70%), followed by pyruvate (glucose/lactate), ketone bodies, and amino acids. In early stages of HF, myocardial fatty acid utilization may be unchanged or augmented. In advanced stages, the myocytes switch from fatty acid to glucose oxidation, returning to a fetal pattern of energy substrate metabolism [48,59,60] (Table 1). Furthermore, myocytes may become insulin-resistant, leading to a decline in glucose/pyruvate utilization (metabolic inflexibility) [60,61,62,63,64,65,66]. Even though glucose uptake is increased, it does not always translate into increased glucose oxidation. Through overexpression of Glut1, the uptake increases glycolysis. Per glucose molecule, glycolysis produces two molecules of ATP compared to 31 molecules of ATP by oxidation. Thus, the energy deficit is not compensated for by substrate switch [115,116].

As described by Ritterhof et al. there is an upregulation of glucose uptake and glycolysis with either no change or even a decrease in glucose oxidation, resulting in uncoupling of substrate uptake and oxidation [59]. Ultimately, this uncoupling reduces cardiac energy availability, the affected heart exhibiting up to 30% less ATP than the healthy one [46,117].

Myocardial ketone body oxidation is increased in HF. Recent studies show this metabolic shift as a key metabolic adaptation in the failing human heart, indicating the potential of ketone bodies as an alternative fuel for HF with reduced and preserved ejection fraction (EF) [35,65,67,68,69,70,71,72,73,74,75]. An increased ketone body oxidation is also seen in RV failure like in pulmonary arterial hypertension (PAH) [118].

*Reduced energy production.* In the failing BV heart, electron transport chain activity is altered (Table 1). Alterations in mitochondrial number, structure and function, in part due to accumulation of reactive oxidative species (ROS) harming mitochondrial deoxyribonucleic acid, may be causes of altered electron transport chain activity [97,119].

*Increase in reactive oxidative species.* Oxidative stress is involved in the development and progression of cardiac remodeling in HF [76]. ROS impair the electrophysiology of the contractile function by denaturing proteins involved in contractility (including L-type calcium channels, sodium channels, potassium channels, and sodium-calcium exchangers [120]) and trigger hypertrophy through modifications in the extracellular matrix [121].

*Insufficient energy transfer to ATP-consuming reactions.* CK energy transfer shuttle works by transferring high-energy phosphate groups from creatine phosphate to ADP to produce ATP. It was the initial mechanism of energy starvation discovered in HF, with creatine deficiency [47]: In HF, the CK system is compromised due to a variety of factors, including decreased levels of CK enzymes and alterations in the composition of the mitochondrial membrane (Table 1). As a result, ATP levels in the heart decrease. Phosphocreatine and total creatine levels decrease by up to 30–70% in an early stage of HF [48,77,78,79]. Consequence is the inability to deliver ATP on increased workload [122]. Reduced CK flux is a significant predictor of HF outcome [117]. Acting on reduced CK flux and on its enzymes is considered a potential HF treatment target [117,123].

*Epigenetic and transcriptional changes: reactivation of fetal gene expression in the failing BV heart.* The activation of fetal cardiac genes which encode proteins involved in contraction, calcium management, and metabolism, is associated with pathological heart hypertrophy. A deterioration in heart function is associated with such reprogramming. Conversely, improvement in cardiac function is associated with normalization of cardiac gene expression in the failing heart [124]. The maturation of fatty acid oxidation was delayed according to a clinical investigation based on RV myocardial biopsies from patients with CHD. Key metabolic enzyme hyperacetylation was prevented by secondary hypertrophy [125]. Lysine acetylation is involved in regulating cardiometabolic diseases. Nicotinamide adenine dinucleotide-dependent deacetylase sirtuin-3 (SIRT3) expression was downregulated in failing hearts from patients with obesity and metabolic syndrome, which led to cyclophilin D hyperacetylation, hyperacetylation, mitochondrial permeability transition pore opening, and cardiac dysfunction [126]. In pressure overload-induced cardiac hypertrophy, histone acetylation is related to inflammation, collagen deposition, and cardiac contractile function [127]. Histone deacetylase inhibition was found to reduce cardiac hypertrophy and fibrosis in spontaneously hypertensive rats by increasing 3-acetylation on the promoters of MR target genes and suppressing gene expression [128]. The HDAC inhibitor valproic acid was shown to improve the development of atrial remodeling and postpone the onset of atrial fibrillation in mice by 4 to 8 weeks [129]. Another HDAC inhibitor, suberoylanilide hydroxamic acid, was discovered to prevent cardiac arrhythmias in dystrophic mice, including QT interval prolongation [130]. Both class I and class II HDAC are inhibited by the HDAC inhibitors that prevent LV hypertrophy.

*Metabolomics as a bridge between gene and metabolism.* Metabolomics is more and more used to analyze metabolites and intermediates in cardiology [131], as well as pediatric cardiology [132,133]. Interpretation can be complex.

Ketolytic metabolism was described as being increased in HF [134]. In atrial fibrillation, elevated blood levels of acylcarnitine were found and discussed as reflective of defective mitochondrial beta-oxidation [135,136].

### 2.3. Right Ventricular Failure: The Case of Pulmonary Arterial Hypertension

In fetal circulation, the right ventricle (RV) is responsible for up to 60% of the cardiac output [137]. Thickness and contractility are likely to be similar between LV and RV at this stage [138]. However, there are deep metabolic and electrophysiological differences between LV and RV, such as differences in systolic Ca^2+^ [139]. The molecular structure of the RV is quite different from that of the LV [140,141]. Metabolic pathways and gene transcription mechanisms in the state of pressure-overload are different in LV and RV [142]. Dysfunction of the RV is accompanied by a release of ROS, combined with chronic nitric oxide deficiency [143]. Additionally, in RV hypertrophy specific mitochondrial remodeling was described [144].

RV failure shares with LV failure the perturbation in substrate consumption. Glucose homeostasis as well as fatty acid metabolism are impaired [82,83,84,145,146,147,148]. Acylcarnitine levels are disrupted in RV failure and have been associated with insulin resistance and adverse outcome [149,150,151,152].

*Untypical substrate consumption*. PAH is a disease resulting in RV failure. Initially regarded an isolated disease of the pulmonary circulation, evidence is accumulating that PAH is to be considered from the metabolic perspective [153,154].

*Abnormal glucose oxidation.* Abnormal glucose metabolism is observed in patients with PAH, even without manifest diabetes mellitus [82,83,84] (Table 1). In Pugh et al. [82], 56% of patients with PAH had an Hb1ac level of over 6.0%, and 15% of patients of over 6.5%—this being undiagnosed DM, reminding of findings in Fontan patients of Ohuchi et al. [155]. Insulin resistance worsened pulmonary phenotype in the West study, implying a possible causal role in PAH [83,85].

*Altered fatty acid oxidation.* Parallel to abnormal glucose metabolism and insulin resistance, alterations of fatty acid oxidation were found in PAH [81]. In plasma, circulating free fatty acids and acylcarnitines are significantly elevated in a similar pattern as found in Fontan patients [43]. In an animal model, altered carnitine function was tied to decreased mitochondrial function and altered nitric oxide signaling [156].

*Increased ketogenesis.* An increased conversion of fatty acids into ketones (ketogenesis) was found to correlate with better clinical health in PAH [118]. This is consistent with the adaptively increased uptake of ketones in HF [69]. Aarhus university is conducting a clinical trial on ketones for PAH patients [86]. There are some rationales behind such an approach. In a previous study, they found a 40% increase in cardiac output under treatment with beta-hydroxybutyrate (BHB) infusion, with an increase in RV function as well as a decrease in pulmonary vascular resistance by 20%. Their published results focus on the hemodynamic effects with an average increase in cardiac output by 27%, and a decrease in pulmonary vascular resistance by 18%, irrespective of the cause of right-sided HF (10 patients with PAH, 10 patients with chronic thromboembolic pulmonary hypertension), and at average a blood ketones level of 3.3 mmol/L [87].

While our article was submitted, a non-reviewed article from the Lillehei heart institute (Minnesota university) describes that compensatory ketosis is absent in RV failure, in contrast to LV failure. The therapeutic stimulation of ketolytic activity is improving RV function, suppresses NLRP3 inflammasome activation and blunts myocardial fibrosis [88]. Those authors hypothesized an RV-liver-axis behind this specific RV dysregulation.

*Oxidative stress.* The electron transport chain in PAH shows pronounced alterations [89,90]. Proteomics studies in PAH identified an increased ROS production that might be related to a loss of antioxidant response [91,92].

*Decreased creatine kinase shuttle.* Alterations in CK were found in diastolic dysfunction in an animal model with RV HF [93,157].

*Alterations in signaling pathways.* Histone deacetylase inhibitors are acting differently in LV, or RV hypertrophy [158,159]. Due to the complex pathogenesis of PAH, more than a single epigenetic modulation to reverse PAH might be required [160].

*Metabolomics findings.* In right-sided HF, metabolomics analysis found specifically elevated blood levels of L-carnitine, acetyl-L-carnitine and long-chain acylcarnitine. Alteration of beta-oxidation of fatty acids increases the concentration of acyl-CoA, thus increasing acylcarnitine levels. The conclusion is that an increased level of acylcarnitine may reflect significant inhibition of the mitochondrial fatty acid beta-oxidation in PAH [161].

### 2.4. Energy Metabolism and the Single Ventricle after Fontan Palliation

*Altered substrate consumption—glucose oxidation.* Pyruvate metabolism might be altered in SV patients, especially in those with HF. One metabolomics study found elevated levels of circulating pyruvate in this group of patients, which might indicate an alteration of pyruvate metabolism and glucose oxidation [95] (Table 1). Noteworthy is that the authors consider pyruvate therapy.

*Altered substrate consumption—fatty acid oxidation.* From biopsies collected during cardiac surgery, it was discovered that cardiac metabolic maturation happened in HLHS (dominant RV) through an increase in AMPK and PPAR-gamma coactivator 1 alpha, and that control of fatty acid oxidation is not impaired in the hearts of HLHS children [162]. This suggests beta-oxidation to work properly. Thus, SV HF might follow a metabolic pattern similar to that of BV HF, exhibiting the reverse Randle effect [105].

Surprisingly, in a very recent metabolomics study functional analysis of the mitochondrial CPT system demonstrated significantly decreased activity of the mitochondrial CPT transporters, suggesting that the diminished myocardial acylcarnitine is related to an overall decreased capacity of the failing SV to oxidize long-chain fatty acids, resulting in a diminished rate of cardiac ATP production [94]. Those findings suggest that due to the decreased enzymatic activity of CPT1/CPT2, beta-oxidation is altered in univentricular hearts which would explain the higher acylcarnitine levels found in Fontan patients compared to healthy controls [43].

Another recent metabolomics study suggests differences in 2-oxoglutarate, isocitric acid, malic acid, and cis-aconitic acid that could reflect alterations of the Krebs cycle [163]. The authors suggest Krebs cycle activation might be necessary to increase cardiac output to counteract hypoxia in Fontan patients. Nevertheless, this interesting finding would require further research, as the study compares metabolomics of various single ventricle architecture to different malformations with inhomogeneous presence or grade of pressure or volume burden such as tetralogy of Fallot or ventricular septum defects. Moreover, the number of patients included is low (*n* = 14), and the study includes patients with severe atrioventricular valve regurgitation or a history of protein-losing enteropathy.

*Induced ketolysis in single ventricle physiology—early reports on the use of Sodium-glucose co-transporter-2 inhibitors.* The decreased activity of CPT2 in SV patients without HF, and CPT1/CPT2 in SV patients with HF [94], as well as elevated circulating level of carnitine [95] might indicate metabolic perturbations. Pires da Silva et al. consider pyruvate therapy [95,164]. This is part of an approach to compensate for the decreased activity of CPT1/CPT2 by therapeutic use of cardiac anaplerosis [165]. An alternative approach is to make use of therapeutic ketosis or induced cardiac ketolysis. One means to stimulate ketone metabolism is the use of sodium-glucose co-transporter-2 (SGLT2) inhibitors as studied in BV HF [166,167,168,169,170,171], an approach supported by a case report (Fontan circulation, 5 patients), where rehospitalization rate was reduced without acute adverse effects [172]. Although based on a very limited patient number, objective data such as increases in systemic oxygen saturation, serum albumin level, and estimated glomerular filtration rate, as well as a decrease in plasma NT-proBNP-level are promising, as NT-proBNP > 100 pg/mL has a 91% sensitivity for significant ventricular dilation, and one of 82% for ejection fraction <50% [173], and is predictive of adverse outcome [174].

While SGLT2 inhibitors’ mechanism still is not completely understood, one of the main hypotheses is that SGLT2 inhibitors are increasing circulating ketone levels [168]. The improvement of parameters seen in the first use of SGLT2 inhibitors on Fontan patients match the effects of ketone bodies on hypoxia [175] and glomerular filtration rate [176], as reported in non-Fontan studies.

*Inflammation and oxidative stress.* In HLHS patients, disorders of cellular respiration are suspected to be present [96]. In an animal model, untypically elevated mitochondrial respiratory capacity was discovered, different from the reduced respiratory capacity typically seen in BV LV HF [96,177]. The cause of this elevation is controversial. Based on the Ohia mouse model, Xu et al., hypothesized a genetic mutation [96]. Other investigators suggested an alteration in the cardiac metabolic maturation [178]. We hypothesize that the unphysiological ventricle switch with its mechanical load changes may trigger metabolic alterations. Irrespective of the cause, such a mechanism is able to trigger an elevation of ROS, known to be one mechanism leading to cardiac hypertrophy [76,97,98]. A recent Proteomics study on young children with an SV lesion (prior to Fontan palliation) showed decreased inflammatory cytokines and increased vascular tone modulators compared to healthy controls before stage 2 palliation, and an increase in these analytes shortly after stage 2 palliation [179]. Interestingly, tissue inhibitor of metalloproteinases-1 or and matrix metalloproteinase 7 levels were associated with greater morbidity, suggesting an important role for regulation of extracellular matrix production.

*Systemic metabolic changes.* Abnormalities in Fontan patients’ glucose and lipid metabolism as well as in the neurohumoral axis have been reported, and this even in ‘stable’ Fontan patients with good exercise capacity and without signs of imminent Fontan failure [19,180,181,182,183,184,185], in part revealing similarities to findings in the BV patient with LV or RV failure. Interestingly, metabolomics studies found that even Fontan patients with good ventricular function and without signs of a Fontan failure exhibited similar lipid metabolic pattern as the BV patient with HF, particularly with respect to alterations in serum cholesterol, lipoprotein, phospholipid, and acylcarnitine concentrations [43]. Moreover, there are first reports on alterations in amino acid pathways, with some key analytes showing altered serum levels (asparagine, histidine, taurine, threonine; amino acid-derived analytes such as dimethylarginine, methionine-sulfoxide or glutamic acid), hinting at inflammation, oxidative stress and endothelial dysfunction, altered cell energy metabolism, and elevated myocardial turnover [42], similar to those found in BV patients with congestive HF.

## 3. Induced Ketosis in Patients with a Failing Biventricular Heart

Initially being an intuition in biochemistry [51], evidence accumulates on the therapeutic role of targeting mitochondrial oxidative metabolism [186], and especially ketone metabolism for the BV LV HF [32]. In the development of HF, cardiac alterations of metabolic processes contribute to a reduction of ATP availability, causing a decline in myocyte contractile function. In advanced HF, ketone metabolism is increased; its nature is discussed to be adaptive [29,102,187]. Nevertheless, therapeutic elevation of ketone bodies may have positive results, not only for cardiometabolic health in general but also systemic health in patients with HF in particular [188]. To date, research in this field is conducted on both LV and RV HF.

*Ketones as a substrate for the failing heart.* Early experimental studies showed that BHB is not only a fuel, but a super fuel for the heart [189,190,191,192,193,194]. Twenty-five percent increased contractility and decreased oxygen consumption were found at 5 mM blood level of ketone bodies [191]. It was discovered that in the healthy heart, ketones do not increase cardiac efficiency. However, ketone fuels are capable of increasing ATP production when the main cardiac fuels—fatty acids and glucose—are deficient [195]. Clinical applications are currently in development, for congestive and acute HF [188,196,197].

*Ketones and oxidative stress.* Oxidative stress is involved in the progression of congestive HF, showing a positive correlation between elevated oxidative stress and myocardial dysfunction [76,121,198,199]. BHB is an endogenous specific inhibitor of class I HDAC. In an experimental study, elevated levels of BHB inhibited HDAC, correlating with changes in transcription including those of the genes encoding oxidative stress resistance factors, conferring substantial protection against oxidative stress with decreased ROS production [200]. Oxidation of ketone bodies contributes to free radical homeostasis [40]. Reduced oxidative stress was also observed in mice using a ketogenic diet [201].

*Ketones and inflammation.* Inflammation is associated with cardiac remodeling and HF [202,203,204]. NLRP3 inflammasome is a new therapeutic target in the treatment of HF [205]. BHB is inhibiting NLRP3 inflammasome [206]. In ketogenic-diet-fed mice, BHB serum levels increased to 0.75–1 mM, and inhibited activation of NLRP3 inflammasome. Overexpression of D-beta-hydroxybutyrate dehydrogenase I enhanced antioxidant enzyme expression and attenuated peroxide-induced apoptosis [39]. In neurons, ketone bodies were shown to decrease mitochondrial production of ROS without affecting the endogenous antioxidant glutathione [207]. Consistently, a low-carbohydrate diet reduced inflammation [208]. High-fat diet elevating BHB was able to reduce inflammation and mitigate HF with preserved EF.

*Ketones and mitochondrial respiratory complex activity*. In an experimental model, ketogenic diet normalized complex I and improved complex II-III activities in rats [209]. This was discussed as originating from providing an alternative substrate as well as through the ketone-mediated downregulated oxidative stress.

*Ketones, myocardial contractility, and ventricular ejection fraction.* Infusion of 3-hydroxybutyrate to patients with HF with reduced EF increased the EF by 8% [80]. At the same time, cardiac output increased by 40%, with a concomitant increase in RV function and decrease of pulmonary vascular resistance by 20% each.

*Ketones, myocardial remodeling, and prevention of cardiac hypertrophy*. Inefficient myocardial fuel consumption can cause pathological hypertrophy [210,211,212,213]. Class I HDAC have been found responsible in the development of pathological cardiac hypertrophy and HF [124,214]. HDAC inhibition was found to be a therapeutic target for cardiac remodeling [215]. Overexpression of BBH dehydrogenase 1 has a protective role regarding resilience to pressure overload-induced cardiac remodeling [39]. In an experimental model, BHB infusion increased histone acetylation in the heart, inhibiting HDAC [200]. In preclinical model HF, elevation of BHB through ketone esters reduced pathologic remodeling [216]. Chronic elevation of BHB in dogs decreased adverse remodeling [38]. In a similar way, strict dietary carbohydrate restriction, causing elevation of ketone levels and decreasing mammalian target of rapamycin expression, suppressed hypertrophy in experimental studies [217,218]. This phenomenon was discussed to have clinical implications. In patients with HF with reduced EF, acute infusions of BHB improved contractility [80].

*Ketones, endothelial function, and vascular resistance.* Keeping pulmonary vascular resistance low is necessary in Fontan circulation [219,220]. In BV patients with HF, endothelial dysfunction, induced by oxidative stress, and elevated vascular resistance contribute to the development of HF and are associated with an increased mortality [221,222]. Mechanisms involved are complex, including oxidative stress, inflammation, and alteration of nitric oxide metabolism. The ketone body BHB presented as a potent vasodilator both in experimental models and human trials [223]. Under BHB infusion, myocardial blood flow increased and induced vasodilation [80,224]. BHB infusion increased blood flow in the renal system [170]. Ketone ester reduced risk of aortic dissection [225].

*Antiarrhythmic potential of ketones.* Ketone oxidation, membrane excitability, and arrhythmogenesis are interrelated. BHB contributed to a 24% improvement in cardiac efficiency, mitochondrial function and the stabilization of cellular membrane potential, enhancing the antiarrhythmic potential of the myocardial cell [36].

*Ketones and oxygen consumption.* Ketone oxidation spares oxygen consumption and is neuroprotective through two mechanisms, oxygenation improvement and decreased blood carbon dioxide [226,227]. Compared to fatty acid oxidation, ketones produce more ATP per molecule of oxygen [228,229].

*Ketones in RV failure*. Nutritional ketosis improved PAH through reversal of the metabolic syndrome [230]. In a similar way, use of the SGLT2 inhibitor empagliflozin prevented the progression of PAH [231]. A clinical trial is currently conducted on the use of an OHB infusion in patients with idiopathic PAH [86] (Table 1).

## 4. Impact of Ketones Apart from That on the Cardiovascular System Relevant to Fontan Circulation

**Figure 1:**
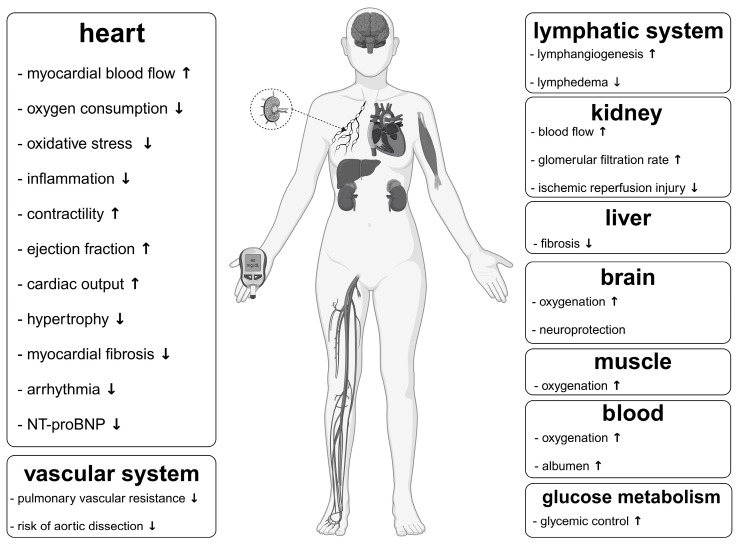
Proven or assumed benefit of ketone bodies on various organ systems. NT-proBNP, N-terminal pro-B-type natriuretic peptide. ↑, increase; ↓, decrease. Created with BioRender.com.

The Fontan circulation with its elevated central venous pressure and limited cardiac output has consequences on all organ systems [19,232]. Ketone bodies are organ-protective. In the following, we aim to estimate to which extent Fontan circulation pathophysiology benefits from this protection (Figure 1).

*Hepatoprotection.* Deficiencies in hepatic ketogenesis are associated with non-alcoholic fatty liver disease and fibrosis [233]. Therapeutic ketosis was explored in the treatment of non-alcoholic fatty liver disease, with effective improvement and reduction of fibrosis [234,235,236,237].

*Renoprotection.* Numerous studies show detrimental effects of the Fontan circulation on the kidneys [238,239,240,241,242]. Renal ketogenesis requires further exploration. Nevertheless, renal ketogenesis is reported to be a mechanism protecting against renal ischemia-reperfusion injury [243,244].

*Neuroprotection.* Mild hypoxia is a common feature of the Fontan circulation [245], and ketones could be a therapeutic strategy to counteract the effects of this hypoxia. For certain anatomical variants of a SV, optimizing oxygen consumption might be critical considering altered coronary perfusion. A ketogenic diet improved the cerebral oxygen level in hypoxia after an epileptic event [175]—the assumed mechanism being altered substrate consumption, reduced glycolysis, and accumulation of lactate [246]. Exogenous ketones increased blood and muscle oxygenation in hypoxia [247].

*Lymphangiogenesis.* Evidence is appearing on the role of ketogenesis on the lymphangiogenesis after corneal injury and myocardial infarction in the BV patient [248]. The mechanism is also on trial for alleviating lymphedema [249,250]. Whether ketone bodies might play a therapeutic role in abnormal lymphatic flow in Fontan circulation requires further research.

*Abnormal glucose patterns*. Abnormal glucose metabolism is one of the underdiagnosed complications of Fontan circulation [155,180]. Alteration of insulin sensitivity is hypothesized to be a factor of Fontan-associated liver disease, which affects a high percentage of Fontan patients [251,252]. Having in mind that ketogenic diet and carbohydrate restriction are established therapies to treat insulin resistance and to improve glycemic control [253], facing the abnormal glucose metabolism inherent to Fontan, the therapeutic potential of ketogenic diet and carbohydrate restriction might be worth considering.

## 5. Rationale of a Targeted Metabolic Therapy in Fontan Patients

Current approaches to HF in Fontan patients are widely based on treatment regimens for BV HF. Their application varies among centers, and polymedication is common, often with the potential of complex drug interaction [22,23]. Apart from the therapeutic potential of ketone bodies in BV HF that should apply to SV patients, there are features unique to Fontan circulation that—based on the effects on similar complications—might benefit from therapeutic ketosis.

**Figure 2:**
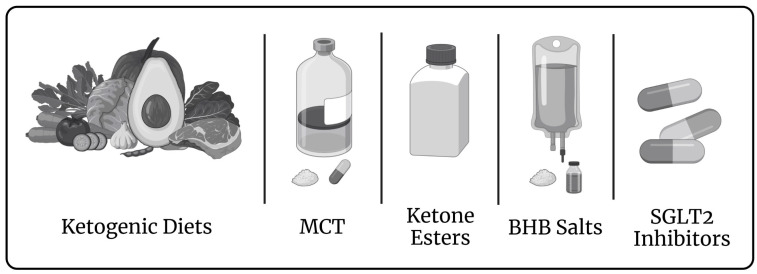
Means to achieve therapeutic ketosis. MCT, medium chain triglycerides; BHB, beta-hydroxybutyrate; SGLT2, sodium-glucose co-transporter 2. Created with BioRender.com.

*Therapeutic modulation of the ketone pathway*. SGLT2 inhibitors are considered to positively address HF through an increase in cardiac ketone oxidation [168]. The first trial of SGLT2 inhibitors on Fontan patients is promising [172]. Though the trial size was limited, the benefits seen in the study support therapeutic ketosis in HF. SGLT2 inhibitor use is currently supported by FDA only for patients over 18 years old [254]. Other means to achieve therapeutic ketosis including ketogenic diet, medium chain triglycerides supplementation, or application of ketone esters (Figure 2).

*A strong case for therapeutic ketosis in Fontan patients.* Therapeutic ketosis has been in use already for 100 years in pediatric neurology [255]. Numerous reviews judge it a safe dietary therapy with minor adverse effects to be monitored [256,257]. It is a sustainable therapy [258], and it is used from toddler age to adult age [259]. The therapy has spread from pediatric neurology to other fields, such as certain inherited metabolic disorders [260], adults’ BV congestive LV HF [216,217,218], intensive care management [261], or oncology [262]. Due to the unique nature of SV patients and the potential of interactions with their polymedication commonly present, induction of ketosis should only be undertaken under close medical control.

*Additional candidate mechanisms for metabolism-targeted therapy for Fontan patients.* Cardiac anaplerosis might be able to replete tricarboxylic acid intermediates and alleviate the substrate consumption alterations [165]. Among other regimens, there is pyruvate therapy [164], application of glutamine [263] (PAH patients showed an increased anaplerosis under glutamine [264]), or branched-chain amino acids [265] and odd-chain fatty acids [266,267]. Octanoate is modulating metabolic acetyl-CoA histone acetylation, promoting cardiac repair after myocardial infarction [268]. It is currently applied to Fontan patients with lymphatic complications such as protein-losing enteropathy (supplementation of the low-fat diet with medium chain triglycerides) [269]. It is unclear how octanoate could bypass CPTI/CPTII transporter and act as a fuel in the mitochondria in the cardiomyocyte, the results being controversial [270,271].

## 6. Limitations

SV malformation is a rare disease. Most studies on cardiac and systemic metabolism of Fontan patients were conducted on a limited number of patients. The etiology of all SV malformations might not be identical, and metabolism as well as gene transcription might vary. Furthermore, metabolomics results should be treated with caution as interpretation can be complex. Large-scale (multicenter) studies are necessary to further explore the metabolic impairment of the SV heart and the ketones‘ therapeutic potential in the respective patients.

## 7. Methods

Our literature review compares the metabolism of patients with an SV circulation after Fontan palliation with that of patients with a healthy BV heart, or different subtypes of a failing BV heart, by Pubmed review of the literature on cardiac metabolism, Fontan failure, heart failure, ketosis, metabolism, published in English from 1939 to 2023.

## 8. Conclusions

Evidence is growing that SV circulation after Fontan palliation not only is a hemodynamically challenging state, but also raises a metabolic issue with its alterations of fatty acid oxidation as well as glucose oxidation, similar to those reported in the failing BV heart, generating metabolic instability and disturbed energy production that *per se* may become a cause of circulatory failure. Evidence accumulates that stimulating ketone oxidation as a targeted metabolic therapy might be a therapeutic strategy to address HF in BV patients. Therapeutic ketosis may be worth considering also in the treatment of Fontan patients, as ketones positively affect not only the myocardial energy metabolism, but also the global Fontan pathophysiology. Induced ketosis seems promising as a therapeutic strategy for chronic ventricular failure and low-grade inflammation, as well as diseased liver, kidney, and intestines. Ketone esters, 1,3 butadeniol, or octanoate might provide a third fuel to the SV heart, and a well-formulated ketogenic diet taking into account the micronutritional status inherent to Fontan patients may have an integrative, concerted effect on the several complications of Fontan circulation.

## Figures and Tables

**Table 1:** State of the heart and metabolic state.

	Metabolic State					
	Substrate Consumption			ETC	CK	Therapeutic Ketosis
	*Fatty* *acids*	*Glucose*	*Ketone bodies*			
**Healthy heart**	60–70% [57,58]	Remaining [57,58]	Remaining [57,58]	balanced		
**HfrEF**	Early HF: no change [48,59,60] Late HF: ↓ [48,59,60]	Early HF: ↑ [48,59,60] Late HF: ↓ [60,61,62,63,64,65,66]	Progressing HF: ↑ [35,65,67,68,69,70,71,72,73,74,75]	Loss of electrons [76] Accumulation of ROS [76]	early HF: ↓ [48,77,78,79]	CO +40% [80] EF +8% [80]
**BV-HF**	↓ [81]	↑ [82,83,84,85]	Progressing HF: ↑ [69,86,87,88]	Loss of electrons [89,90] Accumulation of ROS [91,92]	↓ [89,93]	CO +27% [87] PVR—18% [87]
**SV-HF**	↓ [94]	↓ [95]	?	Loss of electrons [96] Accumulation of ROS [76,97,98]	?	?

BV, biventricular; CK, creatine kinase energy shuttle; CO, cardiac output; EF, ejection fraction; ETC, electron transport chain; HF, heart failure; PVR, pulmonary vascular resistance; ROS, reactive oxygen species; SV, single ventricle; ↑, increase; ↓, decrease. Note the similar findings for BV HF and SV HF with regards to substrate consumption (fatty acids, glucose, ketone bodies). Thus, therapeutic ketosis is worth considering in the treatment of Fontan patients.
